# Safe uncertainty: Reflecting on the pandemic responses of two Asian cities

**DOI:** 10.1177/1473325020973331

**Published:** 2021-03

**Authors:** Timothy Sim

**Affiliations:** The Hong Kong Polytechnic University, Hung Hom, Hong Kong; Singapore University of Social Sciences, Singapore, Singapore

**Keywords:** Culture, uncertainty, race, racism, COVID-19, safety

## Abstract

This reflection focuses on the salient racial, cultural and political processes in the response to COVID-19, particularly in Hong Kong and Singapore, using a framework that examines safety and certainty or the lack of it. It begins by examining the awful racism internationally toward Chinese and the unique Chinese culinary practices that has become a contentious focus in this pandemic. It will then reflect on the meaning and impact of political contexts, with reference to the use surgical masks in Hong Kong and Singapore. Next, it will discuss the disruptions and discoveries for social work teaching and learning and practice during this turbulent time. The reflection will end by looking at the silver linings, and re-thinking about safety and certainty for individuals and social work development, as the pandemic continues to evolve.

There is a Chinese adage, “If you know your enemy, you win every battle” (*zhi-zi-zhi-bi; bai-zhan-bai-sheng).* In relation to COVID-19, however, our knowledge of the enemy is still marked with uncertainty. People are only now beginning to discover the structure of the COVID-19 pandemic, how it spreads in different communities, the clinical features of the disease, potential drug targets, how effective quarantine measures are, the psychological effects of the outbreak on health workers, and so on (Callaway et al., 2020).

## Certainty and safety

**Figure 1.**
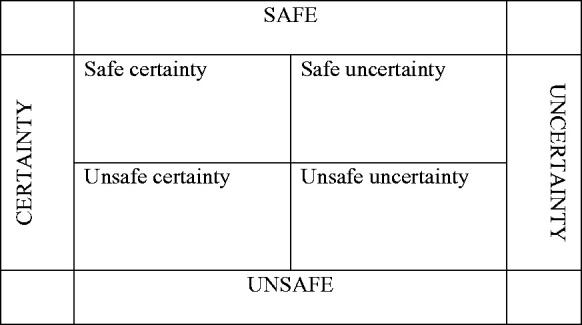
Toward safe uncertainty (Source: Mason, 2019).

The pandemic has heightened our awareness of safety and our sense of uncertainty, as well as the need to protect ourselves, others and our environment in a respectful manner. The two dimensions of Mason’s (2019) framework—safe–unsafe and certainty–uncertainty—entail four possible positions: unsafe uncertainty, safe certainty, unsafe certainty and safe uncertainty. I find this framework highly relevant for social work assessment and practice in times such as those in which we now find ourselves. In situations of unsafe uncertainty, such as those that prevail at the outbreak of a pandemic, there is often a loss of the belief that one can usefully influence events in one’s own life and the lives of significant others. Conversely, occupying a position of safe certainty means that the desire for certainty has been met, often in the form of protective measures imposed from outside (e.g., lockdown measures to deter the spread of COVID-19). Persons occupying a position of unsafe certainty tend to be certain of their points of view and often try to convince others of their correctness (e.g., dogmatic politicians who are certain about what can cure COVID-19). Lastly, the position of safe uncertainty is always evolving: “It is a place where doubt, uncertainty, unhelpful difference, can be safely, if at times uncomfortably, explored as part of developing … more constructive, safer relationships” (Mason, 2019: 347). Such a position is highly relevant to the experience of the pandemic and capable of responding as the situation continues to evolve (Figure 1).

Using Mason’s framework, I reflect on the cultural, political and social contexts and responses to the pandemic of Hong Kong and Singapore, two cities that I have lived in and loved, being a born and bred Singaporean who has worked in Hong Kong for almost two decades.

## Unsafe certainty: Racism and cultural practices

When the virus first emerged in late 2019, it was named after “Wuhan” because the initial outbreak was traced to game meat trading in Wuhan city of Hubei province, China. Some even called it a “Chinese” virus, as they were certain that it was exported from China. The World Health Organization (WHO) renamed the virus “COVID-19” in mid-February 2020. Despite the recommendations of health officials, some global leaders continued to use derogatory terms, prompting a blame game over who was politicizing the pandemic (Rogers, 2020). At a community level, there has been an increase in reports of the racist abuse of Asians around the world since the coronavirus outbreak began. In one incident, three members of an Asian family in Texas, including two children aged six and two, were stabbed (Zheng, 2020). On April 9, 2020, *Nature*, one of the world’s leading multidisciplinary science journals, issued an apology for associating the novel coronavirus with Wuhan and China in its editorial and news coverage (*Nature*, 2020). It appealed for people to stop the stigma around the disease because it was fueling racism and discrimination against Chinese people, and undermining diversity on foreign university campuses, scientific institutions and other sources of scholarship.

Although the evidence that the COVID-19 pandemic was caused by the consumption of game meat is inconclusive, research has suggested that it is possible for infectious diseases to cross the species barrier from wild animals to humans (Volpato et al., 2020). Chinese people are known for their appetite for exotic game meat: in Hong Kong, it is still possible to find snake meat soup being sold in the central business district. However, Chinese are not alone in their consumption of game meat; it is also eaten by people from other parts of Asia, Africa, Europe, the Middle East, the UK and USA. Following the idiom, “one man’s meat is another man’s poison,” it is important that we respect the diversity of culinary cultures. However, we must also go beyond the wall of culture and consider seriously our relationship with nature, and the sustainability of our patterns of consumption and exploitation of Mother Earth. The Chinese government has fast-tracked a decision to prohibit the consumption of wildlife, which is an industry worth at least US$74 billion (Xie, 2020), bringing it into effect in early 2020. The concern now is that this might push the consumption of exotic meat in China underground.

## Safe certainty: Mask or no mask

It was uncanny when, in early January 2020, the Hong Kong government strongly urged Hong Kong people to put on masks to help fight the new coronavirus; just a few months earlier, face masks had been banned due to social unrest. Directions abound on the way that masks should be worn for optimal effect and, given their general mistrust of the government and the hard lessons learned in their previous experience of severe acute respiratory coronavirus (SARS-CoV) in 2003 (World Health Organization, n.d.), people in Hong Kong can be relied upon to wear masks to protect themselves and others. In contrast to the ‘weak’ government of Hong Kong and the bottom-up approach of the Hong Kong people, the Singapore government’s initial position was for Singaporeans to wear masks only when they were unwell. When, coming from Hong Kong, I visited Singapore on March 14 to March 21, 2020, I was shocked to see few people wearing masks. The contrast in vigilance between the two cities was stark. In mid-April, by which time coronavirus cases had reached more than 3,000, the Singaporean government dramatically reversed its earlier stance and made it compulsory to wear a mask outside of one’s home, with a fine of US$212 for non-compliance.

While mask-wearing alone cannot stop the virus from spreading, it has helped Hong Kong, and other East Asian cities in China, Japan and South Korea, to keep the situation in check from the outset. Incidentally, it was only in early June 2020 that the WHO recommended the expanded use of face masks, after five months of debate and review. It is now evident that wearing face masks could help reduce the transmission of diseases caused by coronaviruses, including COVID-19, SARS and MERS. With the issuing of this advice, the wearing of masks is now considered a useful protective measure in a pandemic situation.

At the time of writing (August 1, 2020), Hong Kong was desperately battling its third wave of infection and reported its highest daily number of infection of 125 cases and daily death toll of 6 cases since the pandemic began, with increasing number of older people infected and dying. The official infection tally was 3,396 with 33 related deaths. Business in this enclave has been on a standstill. Singapore was not out of the woods yet and reported 307 new cases on August 1, 2020, with a total of accumulated 52,512 infected cases and 27 deaths, with strict social distancing measures in place even though the number of infected cases in the community have been kept low for an extended period. Unfortunately, the vast majority of people in Singapore who have been infected are foreign workers, mostly from South and Southeast Asian countries, such as Bangladesh and India, who used to live in crowded dormitories where social distancing is impractical (Yeung and Yee, 2020). Veteran diplomat Tommy Koh was among local commentators who called attention to the treatment of migrant workers in Singapore as “not first world but third world” (Lim and Kok, 2020). This is a classic example of inequality spreading the pandemic (Ahmed et al., 2020).

## Unsafe uncertainty: Using a face shield in field work

As is the case in all disciplines, social work classes currently have to be conducted online in Hong Kong. Unlike many other disciplines, however, the particular requirements of social work education (along with nursing and rehabilitation sciences) for small group skill-based modules and field work pose especially great challenges. After much contemplation, and coordination with field work supervisors and practicum sites, our department decided that field work should carry on so students could graduate on time, and that we would “tough it out” as long as front-line agencies were continuing with their services. This decision was also based on the strict regulations for field work training in Hong Kong. Because the paramount concern was for the safety of our students, the university and department provided transparent face shields for students on practicum to put over their faces, in addition to wearing surgical masks. Interestingly, our students were vehemently against the idea, and hardly any of them picked up the face shields. One commented on students’ social media: “How disconnected can the Department academics be?” Their concern was exactly what we have taught them about respect and trust in building relationships. The students, while recognizing that face shields may provide them with additional protection, did not believe that an additional shield would be helpful in facilitating work with clients in need. If it is unsafe, the students would rather not see the clients at all than place a barrier between them. I am proud of the sensitivity and sensibility of our social work students in uncertain times such as these.

Working relationships in social work might never be the same again as we learn to coexist with coronaviruses. Other than the wearing of masks, social distancing and home quarantine are measures that need to be thought through carefully. These measures can be trying for many, especially when other issues of safety and protection arise due to the pandemic. For instance, there has been a greater incidence of family violence against women and children in Hong Kong since the start of the pandemic (Sun, 2020). Moreover, clients with chronic mental or physical health needs, working adults who need to support older people or young children, and family members who have lost their jobs due to the economic downturn, will continue to pose many challenges to a social work profession that may itself be affected by cuts or reduced funding. The safety issues of concern to our profession could cut across the medical, physical, psychological and social domains.

## Safe uncertainty: Silver linings

Koh (2020) highlighted seven silver linings on the dark cloud of the COVID-19 pandemic. The first is the Chinese government’s drastic decision to ban the trade of wild animals for consumption and the eating of game meat. The second is a wake-up call that we truly live in a single interconnected world. The third is a reminder of the importance of public health and of having an effective healthcare system in every country. The fourth is the lesson that political leaders produce better outcomes when they listen to expert advice based on facts, science and reason than when they do not. The fifth is the reinforcement of the importance of social capital. The sixth is a reminder that countries should not depend on a single source for their food supply and should increase their food security (and, I would add, that of basic medical supplies such as masks). The seventh and last is the realization of the extent to which the world has become deeply interdependent economically because of international trade and globalization: in a nutshell, “no one is safe unless the whole world is safe” (Koh, 2020). Beyond these seven, there are many other silver linings on this dark cloud, such as the respite given to our natural environment by lockdowns, and the development of new habits and behaviors due to spending more time at home, such as home-based learning, work and exercise. A further benefit might be better treatment of migrant workers and due recognition for their contributions to society. In this period of coexistence with the virus, while potential vaccines are being developed, it is to be hoped that we take this opportunity to reflect on the silver linings and lessons learnt during this pandemic and sustain those new arrangements and behaviors that have been beneficial to individuals and communities.

## Conclusion

COVID-19 and the pandemics to come respect no one, including royalty and those in power. They know of no context, be it geographical, political, cultural or socio-economic. Nonetheless, despites cutting across lines of race, gender and class, they intersect with the challenging conditions and biases arising from racism and sexism, and associated with poverty and other forms of vulnerability. Hence, vulnerable groups, such as people of a certain race, age or economic status, are more adversely affected (Twigg, 2004). Conversely, it is not difficult to imagine how inequality spreads COVID-19 (Ahmed et al., 2020): what goes around comes around in an interconnected world, and others can infect us as much as we can infect them. Through all of this, what is certain to me is that people who are more able and who have more must reach out to those who are less able and who have less, and that there is no alternative to cooperation in beating this pandemic (*Nature*, 2020).
